# Supplementary material for: Indirect protection from vaccinating children against influenza in households
Source: Nat Commun. 2019 Jan 10;10:106. doi: 10.1038/s41467-018-08036-6 (PMC6328591; doi:10.1038/s41467-018-08036-6)
Supplement: Supplementary file 1 — Supplementary Information [file 41467_2018_8036_MOESM1_ESM.pdf]

Supplementary Information

for 'Indirect protection from vaccinating children against influenza in households'

1 SUPPLEMENTARY METHODS 2

1.1 Model for transmission dynamics in households 2

1.1.1 Overview 3

1.1.2 The probability of the digraph 3

1.1.3 Household transmission 4

1.1.4 The probability of infection from the community 4

1.1.5 Susceptibility component 4

1.1.6 Likelihood function for the digraph 5

1.1.7 Agreement between observed data and digraphs 5

1.2 Inference 5

1.2.1 Priors 5

1.2.2 Algorithms 6

1.2.3 Implementation 8

1.3 Model validation 8

1.4 Model Comparison 9

1.5 Model prediction 9

1.6 Details of trial design 10

2 SUPPLEMENTARY REFERENCES 11

3 SUPPLEMENTARY FIGURES 12

4 SUPPLEMENTARY TABLES 17

1 SUPPLEMENTARY METHODS

1.1 Model for transmission dynamics in households

Every household was randomized to be in either vaccine group or control group, and a child was randomly selected in the household to receive TIV vaccine in the vaccine group (denoted as vaccinated child) or placebo in the control group (denoted as control child). We denoted other household members as contacts of the vaccinated/control child. In the study, there were 3 rounds of sera collection, occurring in pre-season (R1), mid-season (R2) and post-season (R3). In the study plan, all participants provided sera in R1 and R3, and participants in ~33% of randomly selected household provided sera in R2. Each household may have slightly different time of sera collection in each round due to logistic reasons.

For an individual *j* in household *i*, we observed a vector $\left(AT_{ij}^1, AT_{ij}^2, AT_{ij}^3, age_{ij}, vac_{ij}, control_i, hs_i\right)$, where $\mathrm{AT}_{\mathrm{ij}}^k$ was the HAI titer level at round *k*, age_{ij} and vac_{ij} was the age and vaccination status, $control_i$ was the indicator function if the household was randomized in the control group and hs_i was the household size. We defined an HAI titer of <1:20, 1:20-1:40 and >1:40 as low, intermediate and high level of titer respectively.

Infections were defined as a 4-fold or greater rise in at least one paired sera, and therefore we defined a variable y_{ij}, where $y_{ij} = 1$ if $\frac{AT_{ij}^3}{AT_{ij}^1} \geq 4$ when AT_{ij}^2 was not available, and $y_{ij} = 1$ if $\frac{AT_{ij}^2}{AT_{ij}^1} \geq 4$ *or* $\frac{AT_{ij}^3}{AT_{ij}^2} \geq 4$ when AT_{ij}^2 was available.

We excluded the antibody titer results of sera samples from participants reporting vaccination during the study period, because the 4-fold or greater rises from those samples could be the results of vaccination. We assumed that the infection status of these participants was unknown.

We only observed final size data for this study. Therefore, the chains of transmission were not observed and inferring the transmission dynamics in households could be difficult. Hence, we used a direct graph (digraph) approach to estimate the transmission dynamics in households[1,2]. In this approach, we represented the potential chain of transmission in each household as a digraph, and considered those digraphs as

augmented data. Then we jointly inferred the posterior distribution of parameters and the digraphs by using a data augmentation MCMC approach.

1.1.1 Overview

Denote G the digraph representing the potential transmission chain in households, y the observed data, and θ the parameter vector:

$$P(G,\theta|y) \propto P(y|G)P(G|\theta)P(\theta)$$

Here, $P(y|G)$ is an indicator function equal to 1 if the infection status of all participants derived from the digraph G agrees with the observed infection status y. $P(G|\theta)$ is the probability of digraph G given the parameters θ. $P(\theta)$ is the prior density function of the model parameters θ.

1.1.2 The probability of the digraph

Here, we omit the index for household in the notation for simplicity. A household of size *n* is represented by a random directed graph with *n* vertices, each representing a household member. Edges are added to represent possible transmission events. An edge between individual *j* and individual *i* indicates that if individual *j* gets infected, then individual *i* will get infected too. An edge between the community and individual *i* indicates that individual *i* will get infected. Those digraphs can be represented by a matrix[1]. Consider for example the following toy example, where each row represents a potential "source" of transmission while each column represents a potential "recipient":

	Participant 1	Participant 2	Participant 3	Participant 4
Community	1	0	0	1
Participant 1	0	1	0	0
Participant 2	0	0	0	0
Participant 3	1	0	0	0
Participant 4	1	0	0	0

In this digraph, participant 1 and 4 were infected from the community and participant 2 was infected by participant 1. Participant 3 was not infected.

In this approach, the presence of an edge is independent of that of other edges. Therefore, it is possible to observe both an edge from individual *j* to individual *k* and one edge from individual *k* to individual *j*. For each digraph, we can infer the final infection status for every individual in the household. We denoted G_i^j the final outcome for individual *j* in household *i* derived from digraph G_i. The relationship between the digraphs and parameters is presented in the following sections.

1.1.3 Household transmission

Variable v^{jk} indicates the presence of an edge from individual *j* to individual *k*, occurring with a probability:

$$P(v^{jk} = 1|\theta) = 1 - \exp(-\lambda^{jk}(\theta))$$

The formulation of $\lambda^{jk}(\theta)$ is as follows:

$$\lambda^{jk}(\theta) = \{\lambda_{h1}I(hs < 4) + \lambda_{h2}I(hs \geq 4)\} * S_k(\theta),$$

where λ_{h1}, λ_{h2} are parameters that measure the strength of transmission in households of size < 4 and ≥4, respectively, and $S_k(\theta)$ is the susceptibility component for individual *k* described in Section 1.2.3.

1.1.4 The probability of infection from the community

In addition to within household transmission, each of the individual experienced a probability of infection from the community. Variable v^{Ck} indicates the presence of an edge from the community to individual *k*, occurring with probability

$$P(v^{Ck} = 1|\theta) = 1 - \exp(-\lambda^{Ck}(\theta))$$

The formulation of $\lambda^{Ck}(\theta)$ is as follows:

$$\lambda^{Ck}(\theta) = \psi * S_k(\theta)$$

where ψ is a parameter that measures the strength of infection from the community and $S_k(\theta)$ is the susceptibility component for individual *k* described in section 1.2.3.

1.1.5 Susceptibility component

For an individual k, his/her susceptibility was:

$$S_k = \exp\{\beta_1 I(age_k > 18) + \beta_2 I(AT_k \geq 20 \& AT_k \leq 40) + \beta_3 I(AT_k \geq 80) + \beta_4 I(vac_k = 1)\},$$

where $\exp(\beta_1)$ was the relative susceptibility for adults compared with children. $\exp(\beta_2)$ and $\exp(\beta_3)$ were the relative susceptibility for household contacts with intermediate HAI titers (1:20, 1:40) and higher HAI titers (>1:40) comparing with those with HAI titers <1:20. $\exp(\beta_4)$ was the relative susceptibility for vaccinated participants, compared with non-vaccinated participants.

1.1.6 Likelihood function for the digraph

For a given household, the contribution to the likelihood function for the digraph would be as follows:

$$L(G|\theta) = \prod_{j:j=0|y_j=1} \prod_{k \neq j} P(v^{jk} = 1|\theta)^{v^{jk}} P(v^{jk} = 0|\theta)^{1-v^{jk}}$$

Note that j was started from 0 to represent the infection from the community.

We assumed households were independent of each other so that the full likelihood was just the product of all household likelihoods.

1.1.7 Agreement between observed data and digraphs

The second level of the model ensured that the proposed digraph, and hence the potential transmission chain, agreed with the observed data:

$$P(y|G) = \prod_i \prod_j I(G_i^j = y_{ij})$$

where G_i^j the final outcome for individual j in household i derived from digraph G_i.

1.2 Inference

We used a data augmentation MCMC approach to explore the joint posterior distribution of the parameter and digraph space[2]. We outlined the algorithm in the following sections.

1.2.1 Priors

For parameters describing the strength of infection in the community ψ and the strength of transmission in households $\lambda_{h1}, \lambda_{h2}$, the prior distribution was a Uniform(0,10) distribution. Then, (2.5%,97.5%) percentile of the probability of transmission, that is equal to $1 - \exp(-h)$, where h could be ψ, λ_{h1} or λ_{h2}, is (0, 0.9999).

For those parameters that related to the susceptibility, the priors were Normal(-3,3). The (2.5%,97.5%) percentile of the exponential of this prior would be (0.0498, 20.09).

1.2.2 Algorithms

At the initial step, we do the following.

Because there was a small number of missing values for pre-season titers (1%), we first imputed the missing values in antibody titers level by using their observed empirical distribution. We allowed the distribution to be different for children and adults.

As explained in previous studies[1,2], the data augmentation approach was restricted to edges between participants that might potentially have been infected (i.e. with final outcome being infected or unknown). We defined potential edges as edges between participants that might have been infected. We defined a non-edge as the absence of a potential edge.

We started from a full digraph, *i.e.* assuming all potential edges in the digraph were present and hence all participants with unknown infection status were infected and updated the digraph and the unknown status in the MCMC algorithm.

At each MCMC step, we did the following updates:

For the model parameter vector θ, we used a metropolis-hasting algorithm to update each of the parameters individually.

We updated the digraph G and the unknown infection status by first deciding to add a potential edge or delete an edge with equal probability.

To add an edge, we randomly selected a non-edge from all the non-edges (including both household and community edges). Next, we computed the corresponding digraph

and infection status for participants with unknown infection status. It was necessary because addition of an edge may change the infection status of the participants with unknown infection status. For example, in the following digraph:

	Participants 1	Participants 2
Community	0*	0
Participants 1: (unknown infection status)	0	1
Participants 2: (unknown infection status)	0	0

If we proposed to add the edge at 0*, then both participant 1 and 2 would be changed from non-infections to infections. Hence, instead of just comparing the likelihood with this edge and the likelihood without this edge, we needed to compare the likelihood for all household members in this household. Addition of an edge would not change the consistency between digraphs and observed data so no checking was needed.

Suppose the total number of potential edges was A and the number of edges in this step is B. Then the probability that accepting the addition of this edge would be

$$\min\left(1, \frac{L\left(G' \middle| \theta\right)}{L(G|\theta)} * \frac{\frac{1}{B+1}}{\frac{1}{A-B}}\right)$$

where $L(G'|\theta)/L(G|\theta)$ was the likelihood ratio of the current digraph G (without the proposed edge) and the proposed digraph G' (with the proposed edge and updated unknown infection status).

For deletion of an existing edge, we needed to ensure the digraph would be consistent with the observed data and therefore every confirmed infection in the observed data set should have at least one edge from the community or other infected household

members. Otherwise, the deletion would be directly rejected. After checking the consistency, we accepted the deletion with the probability:

$$\min\left(1, \frac{L\left(G'\middle|\theta\right)}{L(G|\theta)} * \frac{\frac{1}{A-B+1}}{\frac{1}{B}}\right)$$

where G' was the digraph with the proposed deletion and updated unknown infection status and G was the digraph without the proposed deletion. Again, re-computing the infection status in the selected household would be necessary because deletion of an edge would change the infection status of household members with unknown infection status.

After updating the digraph, we also updated the missing antibody titers level by using metropolis-hasting algorithm.

1.2.3 Implementation

The chain was run for 200,000 iterations with a burn-in of 100,000 and a thinning of 10. The algorithm was implemented in R with Rcpp package so that C++ could be used. One run of the algorithm for 200000 iterations took about 60 minutes on a desktop with processor: Inter® Xeon® CPU W3565 @3.20GHz.

1.3 Model validation

We used the best fitted model to predict the final size distribution and summarized in Supplementary Table 5. All of the credible intervals can cover the observed number of infections, suggesting that the model fit was adequate.

To assess the performance of our estimation procedure, we performed a simulation study. We simulated 50 data sets with a structure identical to that of the observed data (in terms of age, household structure and the availability of infection status in each round), with parameters equal to their posterior median. We then applied our estimation procedure to the simulated data sets and assessed if parameters could be estimated. The result of simulation was summarized in Supplementary Table 6. We found no important bias and the simulation values were in the 95% credible interval for 44-49 times out of 50 simulations for the 10 parameters in the model. It suggested that

our estimation procedure would be able to provide reasonable estimates of the parameters.

1.4 Model Comparison

Since the likelihood of the observed data is not available in the digraph approach, we used an importance sampling method to estimate it[1,3]. For each household, we simulate 2000 datasets, with parameters drawn from the posterior distribution. Then we compared the observed data and simulated data. The contribution to the likelihood of a household was equal to the proportion of simulated data with infection status that exactly matched the observed data, for all household members. To avoid the problem of 0-valued likelihood, we used the approach developed by Cauchemez et al[1]. We assumed the sensitivity and specificity for diagnosing a case were both 99.99%.

After using the above-mentioned approach to estimate the likelihood of the observed data, the DIC was computed as $2\bar{D} - D(\bar{\theta})$, where D is the deviance, equal to -2*log of likelihood.

1.5 Model prediction

To evaluate indirect protection, we conducted a simulation study with parameters drawn from the posterior distribution. Two vaccine strategies were evaluated:

- Strategy 1: vaccinating one child in each household (as in our trial);
- Strategy 2: vaccinating all children in each household.

and compared to the strategy of "no vaccination".

We simulated 10,000 epidemics in 150000 households with parameters drawn from their posterior distribution. The structure of a simulated household was identical to that of a household randomly drawn in the study. For each infected individual, the source of infection for this individual was determined based on the recorded digraph with the following algorithm:

1) If the individual only had edges from the community only, the source of infection was the community.

2) Else, if the individual only had edges from other infected household members, the source of infection was the household.

3) Else, if the individual was the only one with an edge from the community, the source of infection was the community.

4) Otherwise, the source of infection was inconclusive. For all simulations, the maximum proportion of infections with inconclusive source was 4%. In our primary analysis, half of infections with inconclusive source were assigned to the community and the other half to households. In sensitivity analyses, we considered scenarios where they were all assigned to the community or household sources.

For each strategy, we can compute a probability of infection for a given group and from a specific source (Supplementary Figures 1,2 and 4). For a given group (children or adults) and a given source of infection (household, community or both), the indirect protection due to a vaccine strategy was measured by the relative probability (in term of ratio) of infection in that group and from that source under this vaccine strategy, compared to the probability of infection under no vaccination strategy.

These simulations were repeated for each parameter vector randomly drawn from posterior, hence we were able to derive 95% posterior predictive intervals for each of these relative probabilities that correctly captured the effect of parameter uncertainty on model predictions. These relative probabilities were recomputed using 100000 households and they were basically the same.

1.6 Details of trial design

For estimating direct vaccine efficacy, assuming conservatively that 10% of participants in the control arm would be infected with a prevalent influenza strain[4], inclusion of 800 subjects would have 75% power to detect a vaccine efficacy of ⩾50%, with a 5% type I error rate. An unbalanced randomization scheme was proposed, where more participants were included in the intervention arm to enhance acceptability. For estimating indirect vaccine efficacy, recruiting the 2,000 (assuming an average household size of 3.5 = 1 participant + 2.5 household contacts) household members of the 800 study participants (1,200 in intervention arm, 800 in control arm) was anticipated to be sufficient to ensure high power to identify indirect vaccine effectiveness values of interest. While the infection risk of influenza in adults is typically lower than in children, we anticipated an attack rate of 5% in the control arm. The within-household correlation in infection risks was reported as 29% in a previous

study[5]. Allowing for within-household correlation, our study would have 77% power to detect indirect vaccine effectiveness of 40% or greater, with a 5% type I error rate.

Randomization lists were prepared by a biostatistician (V. J. F). Eligible study participants were randomly allocated to the TIV or placebo group at a ratio of 3:2 using a random number generator (R software). A block-randomization sequence was generated, with randomly permuted block sizes of 5, 10, and 15. Blinding of households and study nurses was achieved by identical repackaging of TIV/placebo into numbered syringes by a trained nurse not involved in vaccine administration. A research assistant who had no access to the randomization list allocated unique numbers to participating households based on their order of attendance and these were subsequently matched to vaccine packages. Allocation of TIV/placebo was concealed to participating households, study nurses, and laboratory staff, and was revealed to investigators only after completion of follow-up.

2 SUPPLEMENTARY REFERENCES

1 Cauchemez, S. *et al.* Determinants of influenza transmission in South East Asia: insights from a household cohort study in Vietnam. *PLoS Pathog* **10**, e1004310, doi:10.1371/journal.ppat.1004310 (2014).

2 Demiris, N. & O'Neill, P. D. Bayesian inference for stochastic multitype epidemics in structured populations via random graphs. *Journal of the royal Statistical Society Series B* **67**, 731-745 (2005).

3 Liu, J. S. *Monte Carlo strategies in scientific computing*. (Springer-Verlag, 2001).

4 Hoberman, A. *et al.* Effectiveness of inactivated influenza vaccine in preventing acute otitis media in young children: a randomized controlled trial. *JAMA* **290**, 1608-1616, doi:10.1001/jama.290.12.1608 (2003).

5 Viboud, C. *et al.* Risk factors of influenza transmission in households. *Br J Gen Pract* **54**, 684-689 (2004).

3 SUPPLEMENTARY FIGURES

Supplementary Figure 1. The probability of infection for child contacts and adult contacts for no vaccination, vaccine strategy 1 (vaccinating one child in each household) and vaccine strategy 2 (vaccinating all children in each household). For each panel, the title was in the form of vaccine strategy, group of individuals. It was conducted under the assumption that half of the infections with inconclusive source was infected from the community and another half was infected from the household. 95% posterior predictive intervals are constructed with 10000 simulated epidemics based on the estimated posterior distribution of model parameters (Supplementary Methods).

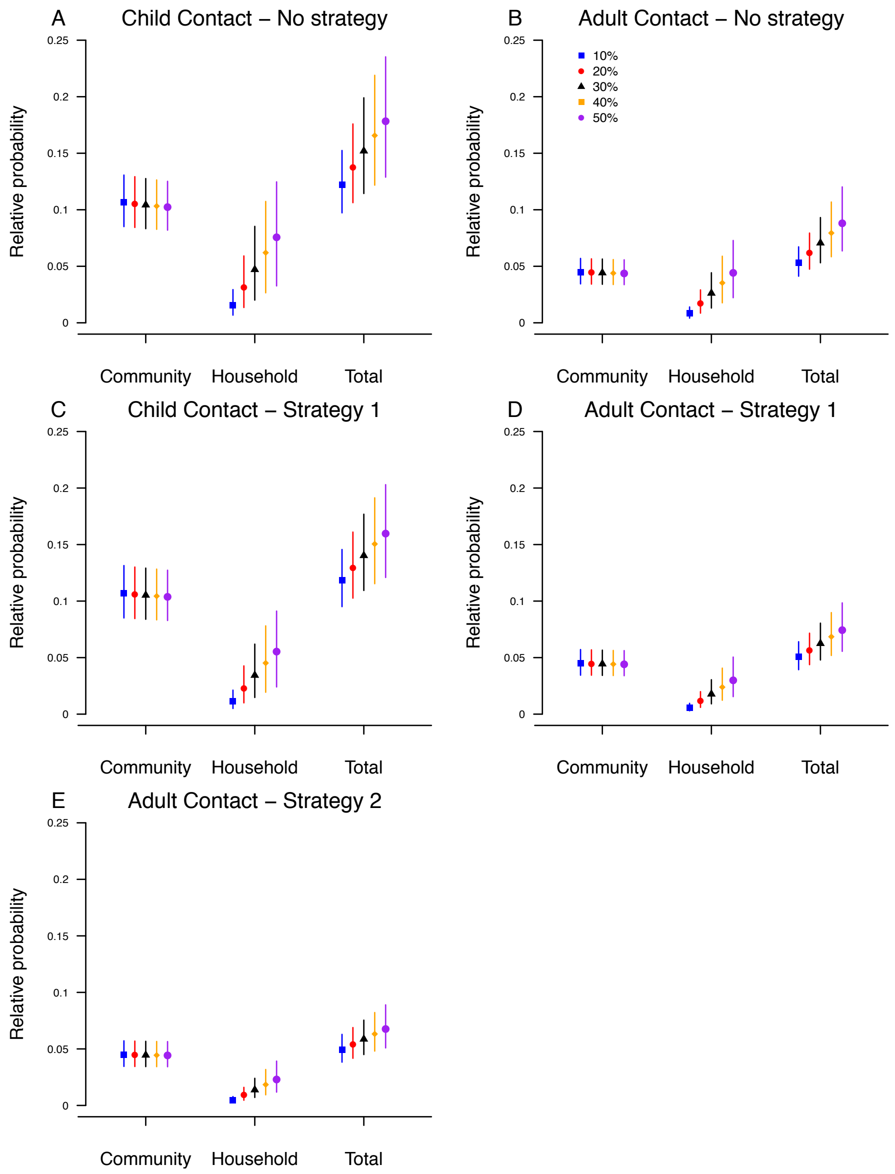

Supplementary Figure 2. The probability of infection for child contacts and adults contacts for no vaccination, vaccine strategy 1 (vaccinating one child in each household) and vaccine strategy 2 (vaccinating all children in each household). The format was the same as Supplementary Figure 1 but it was conducted under the assumption that all infections with inconclusive source were infected from the community. 95% posterior predictive intervals are constructed with 10000 simulated epidemics based on the estimated posterior distribution of model parameters (Supplementary Methods).

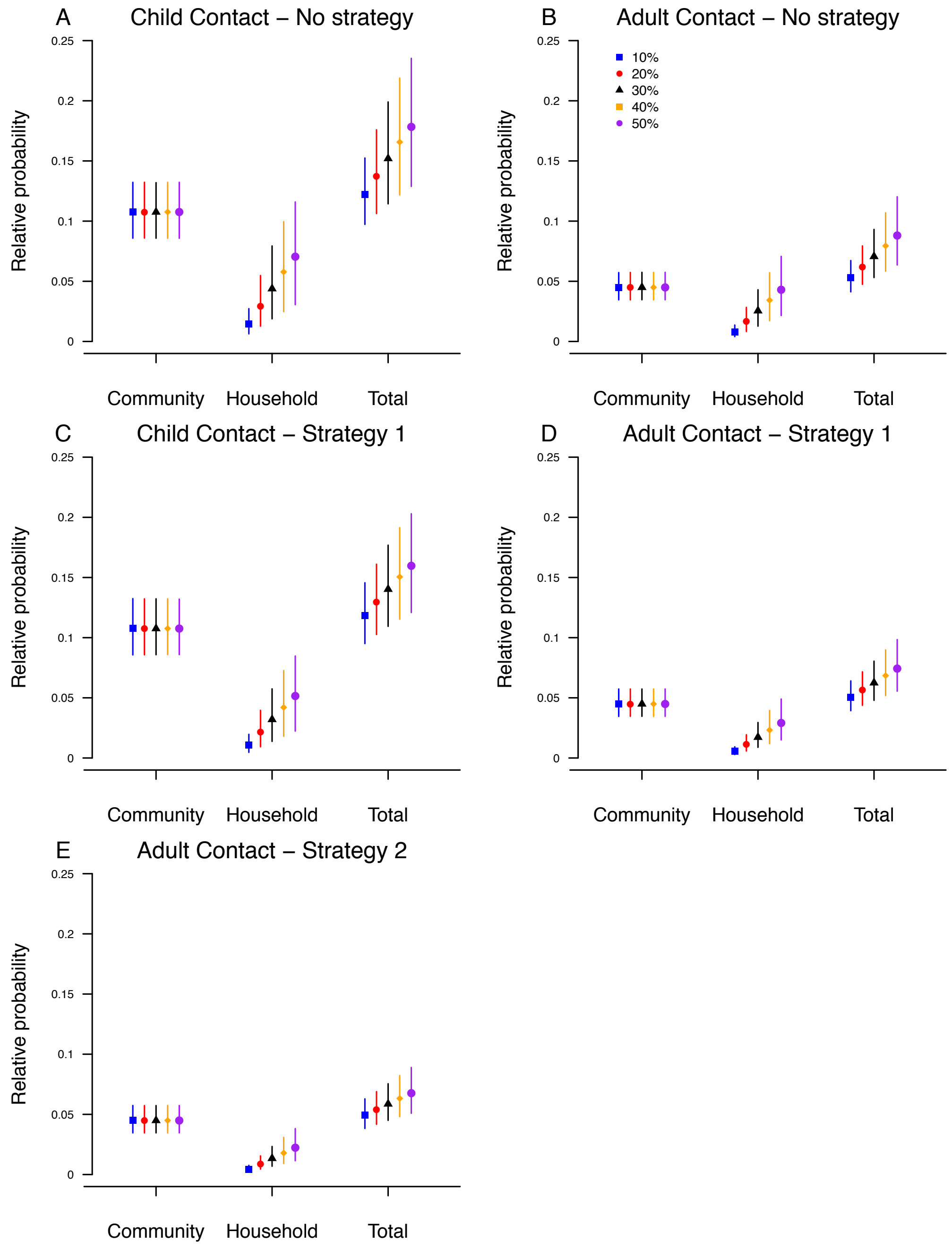

Supplementary Figure 3. The relative probability of infection for household contacts of vaccinated children, under two vaccination strategies (strategy 1: vaccination one child per household; strategy 2: vaccinating all children in the household), compared with no vaccination strategy. The format was the same as Figure 5 but it was conducted with the assumption that all infections with inconclusive source were infected from the community. 95% posterior predictive intervals are constructed with 10000 simulated epidemics based on the estimated posterior distribution of model parameters (Supplementary Methods).

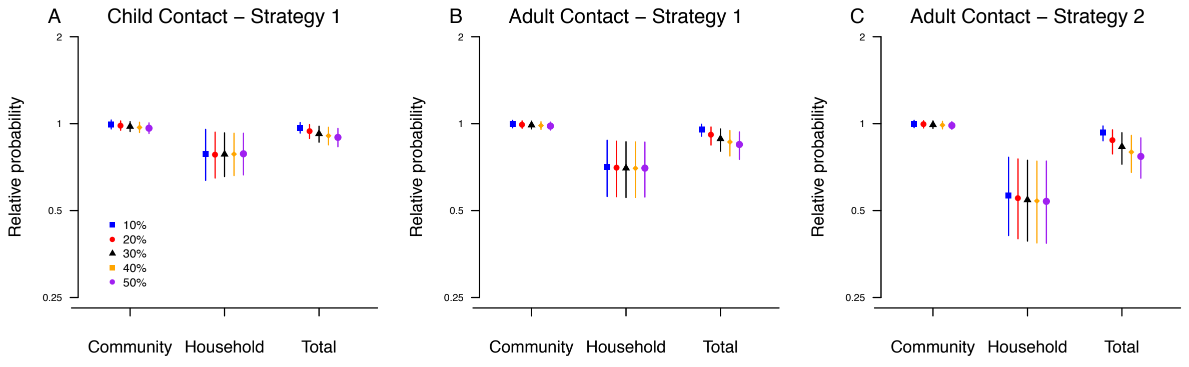

Supplementary Figure 4. The probability of infection for child contacts and adults contacts for no vaccination, vaccine strategy 1 (vaccinating one child in each household) and vaccine strategy 2 (vaccinating all children in each household). The format was the same as Supplementary Figure 1 but it was conducted with the assumption that all infections with inconclusive source were infected from the household. 95% posterior predictive intervals are constructed with 10000 simulated epidemics based on the estimated posterior distribution of model parameters (Supplementary Methods).

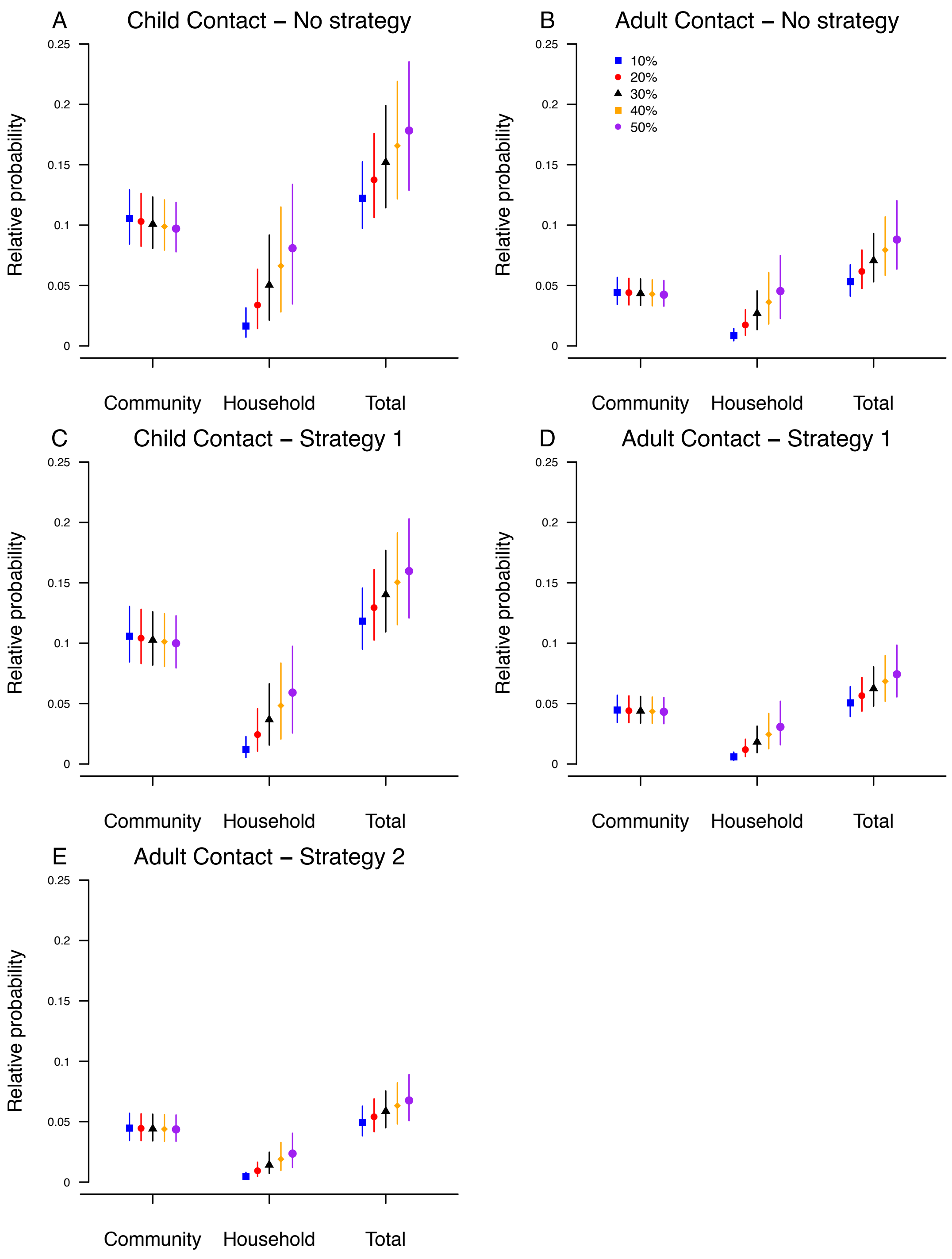

Supplementary Figure 5. The relative probability of infection for household contacts of vaccinated children, under two vaccination strategies (strategy 1: vaccination one child per household; strategy 2: vaccinating all children in the household), compared with no vaccination strategy. The format was the same as Figure 5 but it was conducted with the assumption that all infections with inconclusive source were infected from the household. 95% posterior predictive intervals are constructed with 10000 simulated epidemics based on the estimated posterior distribution of model parameters (Supplementary Methods).

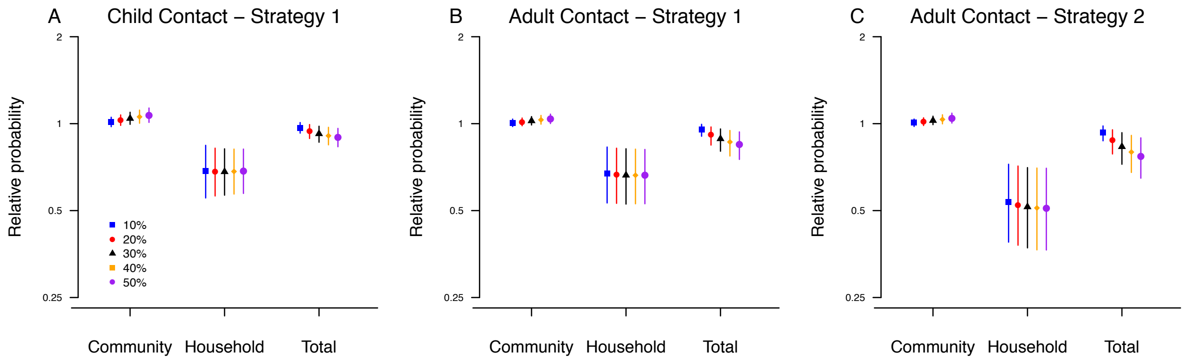

4 SUPPLEMENTARY TABLES

Supplementary Table 1. Characteristics of household contacts in the vaccine trial

Characteristic	Placebo group	Vaccine group
**No. of participants**	776	1146
**Age**		
**⩽18 years**	159 (20.5%)	237 (20.7%)
**>18 years**	617 (79.5%)	909 (79.3%)
**Male**	335 (43.2%)	502 (43.8%)
**Vaccination**	82 (10.6%)	143 (12.5%)
**Serum available in**		
**Round 1**	758 (97.7%)	1129 (98.5%)
**Round 2**	276 (35.6%)	419 (36.6%)
**Round 3**	714 (92%)	1041 (90.8%)

Supplementary Table 2. Demographic information for participants in each round

	Round 1+2		Round 2+3		Round 1+3[1]	
	TIV group	Placebo group	TIV group	Placebo group	TIV group	Placebo group
Number of pairs	424	287	424	287	722	489
Age ⩽18 years	91/424 (21%)	59/287 (21%)	91/424 (21%)	59/287 (21%)	146/722 (20%)	100/489 (20%)
Age >18 years	333/424 (79%)	228/287 (79%)	333/424 (79%)	228/287 (79%)	576/722 (80%)	389/489 (80%)
Male	188/424 (44%)	121/287 (42%)	188/424 (44%)	121/287 (42%)	314/722 (43%)	214/489 (44%)

[1]only participants with missing serum in Round 2 were included in this group

Supplementary Table 3. Timing of sera collection among participants

	Round 1+2		Round 2+3		Round 1+3[1]	
	TIV group	Placebo group	TIV group	Placebo group	TIV group	Placebo group
First serum						
Range	Aug 15, 2009 to Jan 15, 2010	Aug 15, 2009 to Jan 9, 2010	Apr 2, 2010 to May 1, 2010	Apr 2, 2010 to May 1, 2010	Aug 15, 2009 to Jan 23, 2010	Sep 11, 2009 to Feb 6, 2010
Median	Oct 17, 2009	Oct 17, 2009	Apr 16, 2010	Apr 10, 2010	Oct 31, 2009	Oct 30, 2009
Second serum						
Range	Apr 2, 2010 to May 1, 2010	Apr 2, 2010 to May 1, 2010	Oct 1, 2010 to Nov 27, 2010	Oct 1, 2010 to Nov 27, 2010	Sep 11, 2010 to Nov 27, 2010	Oct 1, 2010 to Nov 27, 2010
Median	Apr 16, 2010	Apr 10, 2010	Oct 23, 2010	Oct 23, 2010	Oct 30, 2010	Oct 29, 2010
Days between the date of collections: Median (Range)	182 (92, 257)	182 (98, 259)	196 (160, 238)	196 (160, 238)	370 (265, 420)	364 (273, 427)

[1]Only participants with missing serum in Round 2 were included in this group

Supplementary Table 4. Observed number of infections, defined by 4-fold or greater rise for at least one paired sera.

	Group	Round 1+2	Round 2+3	Round 1+3	All
**0-18 yrs**	**TIV**	7/84 (8%)	3/82 (4%)	7/113 (6%)	17/194 (9%)
	**Control**	9/50 (18%)	2/52 (4%)	10/78 (13%)	20/128 (16%)
**>18 yrs**	**TIV**	18/293 (6%)	9/285 (3%)	15/434 (3%)	42/717 (6%)
	**Control**	10/201 (5%)	5/196 (3%)	10/308 (3%)	25/500 (5%)
**Overall**	**TIV**	25/377 (7%)	12/367 (3%)	22/547 (4%)	59/911 (6%)
	**Control**	19/251 (8%)	7/248 (3%)	20/386 (5%)	45/628 (7%)

[1]Only participants with missing serum in Round 2 were included in this group

Supplementary Table 5. Observed and expected final size distribution in households

		Numbers of infections						
		Household contacts of vaccinated children						
		0	1	2	3	4	5	6
Numbers of household contacts	1	55 - 56.5 (53, 59)	4 - 2.5 (0, 6)	NA	NA	NA	NA	NA
	2	175 - 177.6 (170, 185)	16 - 14.8 (8, 22)	3 - 1.5 (0, 4)	NA	NA	NA	NA
	3	128 - 124.4 (115, 132)	17 - 20 (13, 28)	2 - 2.4 (0, 6)	0 - 0.2 (0, 1)	NA	NA	NA
	4	28 - 28.9 (24, 33)	7 - 5.9 (2, 10)	0 - 1.1 (0, 3)	1 - 0.2 (0, 1)	0 - 0 (0, 0)	NA	NA
	5	6 - 5.3 (3, 7)	1 - 1.3 (0, 4)	0 - 0.3 (0, 2)	0 - 0 (0, 1)	0 - 0 (0, 0)	0 - 0 (0, 0)	NA
	6	3 - 2 (1, 3)	0 - 0.6 (0, 2)	0 - 0.3 (0, 1)	0 - 0.1 (0, 1)	0 - 0 (0, 0)	0 - 0 (0, 0)	0 - 0 (0, 0)
		Household contacts of control children						
Numbers of household contacts	1	36 - 36.2 (33, 38)	2 - 1.8 (0, 5)	NA	NA	NA	NA	NA
	2	111 - 109.3 (102, 115)	10 - 11.3 (6, 18)	1 - 1.4 (0, 4)	NA	NA	NA	NA
	3	87 - 93 (85, 100)	22 - 15.1 (8, 23)	1 - 1.8 (0, 5)	0 - 0.1 (0, 1)	NA	NA	NA
	4	21 - 18.7 (15, 22)	1 - 3.6 (1, 7)	1 - 0.6 (0, 2)	0 - 0.1 (0, 1)	0 - 0 (0, 0)	NA	NA
	5	4 - 4.2 (2, 6)	1 - 1.3 (0, 3)	1 - 0.4 (0, 2)	0 - 0.1 (0, 1)	0 - 0 (0, 0)	0 - 0 (0, 0)	NA
	6	0 - 0 (0, 0)	0 - 0 (0, 0)	0 - 0 (0, 0)	0 - 0 (0, 0)	0 - 0 (0, 0)	0 - 0 (0, 0)	0 - 0 (0, 0)

Each element of the table has the format "observed frequency – expected (posterior mean) frequency (95% Credible interval).

Supplementary Table 6. Simulation study for the model for estimating the cumulative incidence of infection

parameter		Simulation value	Mean	Number of credible intervals that cover the simulation value (out of 50)
Parameter describing the probability of infection in the community	$\boldsymbol{\psi}$: for the whole study period	0.13	0.14	43
Parameters describing the probability of transmission in households	$\boldsymbol{\lambda_{h1}}$: strength of transmission in households with size less than 4	0.2	0.24	47
	$\boldsymbol{\lambda_{h2}}$: strength of transmission in households with size equal to 4	0.07	0.08	44
Relative susceptibility (Ref group: children)	$\boldsymbol{\beta_1}$: adults	0.39	0.35	45
Relative susceptibility (Ref group: low level of titers)	$\boldsymbol{\beta_2}$: intermediate level of titers	0.48	0.42	48
	$\boldsymbol{\beta_3}$: high level of titers	0.42	0.35	50
Relative susceptibility (Ref group: non-vaccination)	$\boldsymbol{\beta_4}$: vaccination	0.29	0.25	46
